# Progress in Global Surveillance and Response Capacity 10 Years after Severe Acute Respiratory Syndrome 

**DOI:** 10.3201/eid1906.130192

**Published:** 2013-06

**Authors:** Christopher R. Braden, Scott F. Dowell, Daniel B. Jernigan, James M. Hughes

**Affiliations:** Centers for Disease Control and Prevention, Atlanta, Georgia, USA (C.R. Braden, S.F. Dowell, D.B. Jernigan);; Emory University, Atlanta (J.M. Hughes)

**Keywords:** SARS, Hotel Metropole, IHR 2005, superspreading events, International Health Regulations, coronavirus, viruses, severe acute respiratory syndrome, China, Canada, WHO, World Health Organization, pandemic, respiratory infections

## Abstract

Ten years have elapsed since the World Health Organization issued its first global alert for an unexplained illness named severe acute respiratory syndrome (SARS). The anniversary provides an opportunity to reflect on the international response to this new global microbial threat. While global surveillance and response capacity for public health threats have been strengthened, critical gaps remain. Of 194 World Health Organization member states that signed on to the International Health Regulations (2005), <20% had achieved compliance with the core capacities required by the deadline in June 2012. Lessons learned from the global SARS outbreak highlight the need to avoid complacency, strengthen efforts to improve global capacity to address the next pandemic using all available 21st century tools, and support research to develop new treatment options, countermeasures, and insights while striving to address the global inequities that are the root cause of many of these challenges.

Ten years have elapsed since the World Health Organization (WHO) issued its first global alert for an unexplained illness, which it named severe acute respiratory syndrome (SARS) (*1*). A few days later, the Institute of Medicine (IOM) released a report, Microbial Threats to Health, that highlighted many of the issues and challenges raised by SARS (*2*). This anniversary provides us with an opportunity to reflect on the international response led by WHO to this new global microbial threat, a response that resulted in control of the pandemic that resulted in >8,000 cases and nearly 800 deaths in >30 countries and had a large economic impact (*3*). The series of emerging and reemerging disease threats since 2003, from avian influenza (H5N1, H7N9) to extensively drug-resistant tuberculosis to the recently recognized novel coronavirus, reinforce the need to avoid the complacency that typically occurs in the aftermath of a successful response to a crisis resulting from an emerging microbial threat.

## Lessons of SARS

Many features of the SARS epidemic and the public health response are worth recalling because they provide reminders of challenges posed by the emergence of a new disease that is transmissible from person to person. Some of these features include the initial lack of field investigative capacity, reference laboratory testing, and reporting transparency from southern China, which resulted in a 3-month delay in the reporting of the severe unexplained illness to WHO; the important role played by an alert clinician in Hanoi, Vietnam, in the initial recognition and response to the illness; the rapid spread of illness to >30 countries; and the effects on health care workers and family members, who were most at risk for person-to-person spread of the infection. Reviewing the events that occurred during the SARS epidemic is an opportunity to highlight the ultimate success of early patient isolation, contact tracing, quarantine, and infection control measures; the importance of rigorous attention to biosafety in laboratory settings; the effects of stigmatization of affected groups; the economic impact as a result of major disruptions in international travel and commerce; the identification of the mode and circumstances of cross-species transmission; and the role of “superspreaders” and superspreading events in the rapid dissemination of the illness. 

In addition, during the epidemic, the leadership provided by WHO facilitated timely exchange of new information among clinicians, epidemiologists, and laboratory investigators around the world. These efforts included the formation of a global network (*4*) of virology and pathology laboratories that used modern diagnostic methods, which contributed to the rapid identification, characterization, and sequencing of the agent and the timely dissemination of critical information and guidance through agency reports, expedited peer-reviewed publications (*5*–*9*), lay media, and the Internet. These experiences exemplify the characteristic features of the global SARS outbreak.

## Emergence in Guangdong Province

Details are sketchy about the earliest phase of SARS as it spread in southern China, but the best retrospective analyses show that the initial cases and clusters occurred in mid-November 2002; its spread to health care workers and family members was a critical aspect of the amplification of the epidemic during January 2003 (*10*). Initial investigations were conducted by provincial public health authorities who did not recognize or failed to report the potential global implications of the epidemic, and initial laboratory investigations incorrectly focused on a possible *Chlamydia* spp.–like organism as the etiologic agent (http://english.peopledaily.com.cn). Once the public health implications were recognized, however, the subsequent response to SARS by China was among the most aggressive and effective worldwide and included substantial improvements in epidemiologic training, laboratory capacity, and mandatory reporting, as detailed below.

## Superspreading Events Linked to the Hotel Metropole

Several superspreading events contributed to the dissemination of the virus. Some of the most dramatic examples included those associated with the Hotel Metropole in Hong Kong (*11*), the Amoy Gardens apartment complex in Hong Kong (*12*), Air China flight 112 from Hong Kong to Beijing (*13*), and an acute care hospital in Toronto, Ontario, Canada (*14*). The episode at Hotel Metropole that contributed greatly to the initial cross-border spread of the disease was particularly noteworthy.

**Figure 1:**
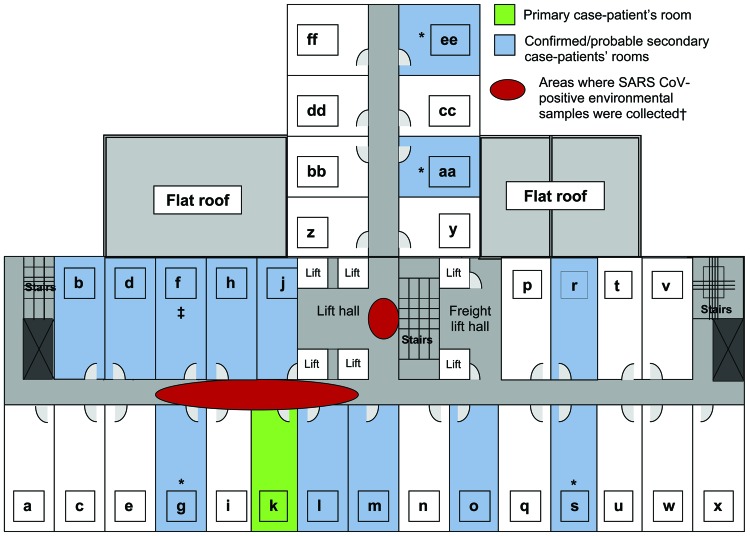
Layout of ninth floor of Hotel Metropole, where superspreading event of severe acute respiratory syndrome (SARS) occurred, Hong Kong, 2003. *2 cases in room; †see (*16*); ‡case-patient visited room. CoV, coronavirus.

The cluster of SARS cases at Hotel Metropole in Hong Kong in 2003, the first superspreading event recognized outside mainland China, was responsible for the spread of the epidemic from Guangdong Province to Canada, Vietnam, Singapore, and Hong Kong itself. In addition to the first 13 cases originally associated with the Hotel Metropole (*11*), a follow-up cohort study of guests from Canada, Germany, England, and the United States who stayed at the hotel concurrent with the index case-patient, a physician from Guangdong, identified an additional 7 cases that met the probable (*2*) or confirmed (*5*) case definition for SARS coronavirus (CoV) infection (*15*). All 20 cases were associated with transmission of SARS CoV on the ninth floor of the hotel, where the index case-patient had stayed for 1 night before becoming critically ill and being admitted to a local hospital the next day. Three deaths occurred among hotel guests who had been identified as case-patients, resulting in a case-fatality ratio of 15%. Known secondary SARS cases were associated with at least 13 (42%) of 31 guest rooms on the ninth floor (Figure 1).

The high rate of infection among guests staying on the ninth floor at the Hotel Metropole is remarkable because they did not have direct contact with the index case-patient. For example, 1 resident of Hong Kong who visited a friend on the ninth floor (but was not a hotel guest) likely acquired his infection during his visit; this person subsequently infected 143 people at Prince of Wales Hospital in Hong Kong (*17*). Epidemiologic evidence suggested an environmental route of SARS CoV transmission. Indeed, environmental contamination with SARS CoV RNA was identified on the carpet in front of the index case-patient’s room and 3 nearby rooms (and on their door frames but not inside the rooms) and in the air intake vents near the centrally located elevators (*17*). Guest rooms had positive air pressure relative to the corridor, and there was no direct flow of air between rooms. The lack of air flow between rooms and the absence of SARS CoV RNA detected inside guest rooms suggest that secondary infections occurred not in guest rooms but in the common areas of the ninth floor, such as the corridor or elevator hall. These areas could have been contaminated through body fluids (e.g., vomitus, expectorated sputum), respiratory droplets, or suspended small-particle aerosols generated by the index case-patient; other guests were then infected by fomites or aerosols while passing through these same areas. Efficient spread of SARS CoV through small-particle aerosols was observed in several superspreading events in health care settings, during an airplane flight, and in an apartment complex (*12*–*14*,*16*–*19*). This process of environmental contamination that generated infectious aerosols likely best explains the pattern of disease transmission at the Hotel Metropole.

The compilation of data from multiple superspreading events in the SARS epidemic yields valuable findings that could be relevant for other respiratory infections of pandemic potential. These events underscore the potential for aerosol transmission in non–health care settings and the dramatic role such transmission can play in the global transmission of respiratory diseases.

## Recognition and Reporting from Hanoi

One of the guests at the Hotel Metropole, a business traveler, was hospitalized at the French Hospital in Hanoi. Called to the investigation of the subsequent illnesses of health care workers at the hospital was Dr Carlo Urbani, a WHO physician specializing in parasitology who was known for having the mindset of an alert clinician and a strong dedication to the principles of public health. In a series of emails from Hanoi to his colleagues at WHO, Dr Urbani sent some of the first messages of alarm and detailed descriptions of the clinical features of what would come to be known as SARS. His reports would lead to an aggressive response by the government of Vietnam, which quarantined the hospital staff and ultimately contained the epidemic there (*20*). It also raised the alarm with colleagues at WHO and the US Centers for Disease Control and Prevention, who would work to characterize and contain the global epidemic. Dr Urbani himself became infected and was hospitalized in Bangkok, where he insisted on obtaining repeated samples from his own respiratory tract, which provided some of the first isolates of the novel CoV (*6*,*9*). Dr Urbani died on March 29, 2003 (*21*), one of many health care workers who responded to those in need, only to become victims themselves.

## Health Care–associated Transmission in Toronto and Taiwan

**Figure 2:**
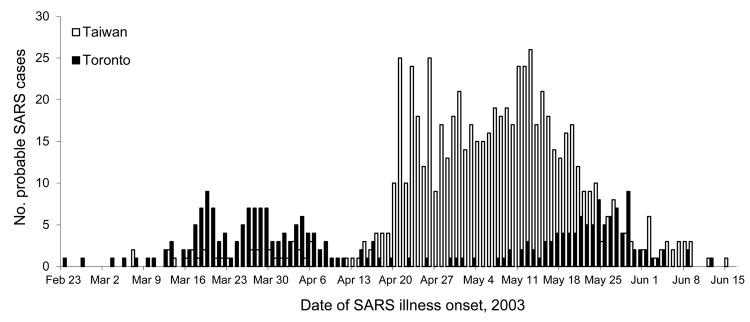
Probable cases of severe acute respiratory syndrome, by location and date of illness onset, Toronto, Ontario, Canada, and Taiwan, February 23–June 15, 2003.

Health care facilities played a substantial role throughout the SARS outbreak as sites of efficient transmission that led to acceleration of disease in communities. These facilities also served a critical role in stopping SARS through strict implementation of infection control practices. Important lessons regarding the epidemiology and control of SARS are evident in the spread of health care–associated SARS in Toronto and Taiwan (*22*). In both places, the spread of SARS was initiated by unrecognized transmission of the virus in health care facilities; however, the outbreaks progressed differently (Figure 2).

The first cases of SARS in Toronto occurred very early in the global outbreak. A 78-year-old woman who had stayed at the Hotel Metropole in Hong Kong in late February 2003 returned to Toronto before dying at home. However, her son had been infected and was subsequently admitted to a Toronto hospital, where nosocomial transmission led to >100 cases among patients, health care workers, and visitors. Prompt institution of practices to control airborne, contact, and droplet infection led to an apparent cessation of transmission, and on May 14, WHO declared that Toronto was no longer a SARS-affected area. Control recommendations were relaxed, and the crisis appeared to have ended; however, unrecognized infection continued among a small number of patients and visitors. Eventually, transmission to health care workers, patients, and visitors resurged, leading to an additional 79 cases, as evident in the bimodal shape of the epidemic curve. After strict infection control practices and vigilance for SARS were reinstituted, the last case was recognized in mid-June, and no other cases were recorded thereafter.

The experience in Taiwan was very different. Soon after the novel CoV was recognized in Hong Kong, officials in Taiwan instituted rigorous port entry screening and isolation among returning travelers who had suspected SARS and their contacts. Public health and academic medical officials focused exhaustive efforts on accurately diagnosing cases of SARS in travelers. This approach appeared to work well for 6 weeks, suggesting that SARS could be prevented from entering the island. However, despite these measures, unrecognized transmission of SARS began occurring in the community. SARS in a hospital laundry worker at the large urban Ho Ping Hospital in Taipei led to exposure of staff and patients and ignited an explosive outbreak that spread to other hospitals and the community. To contain the transmission, patients, staff, and visitors were quarantined in the facility, an action that had rarely been invoked in modern times. More than 1,000 persons were quarantined; some tossed soft drink bottles from windows with protest messages, others communicated the disarray within the facility through cell phone messages, and a few escaped. Public health officials rapidly pivoted in control policies and community response actions to prevent a potential emerging infectious disease catastrophe. The epidemic curve for Taiwan reveals the very rapid rise in cases resulting from the hospital outbreak. Strict infection control practices were mandated in all health care settings. SARS evaluation centers (“fever clinics”) were constructed outside hospital emergency departments. Community use of face masks, fever checks on entry to commercial establishments, and extensive community outreach and education were used to mitigate the effects of SARS. After 2 months of epidemic spread, leading to >600 cases, SARS was eventually contained, and no further cases were reported.

## The Legacy of SARS

After the emergence of SARS, many after-action reports were written, many recommendations were made, and many steps were taken in response to lessons learned. SARS was frightening and had a dramatic effect on global travel and business. The outbreak showed how rapidly a new, fatal pathogen could spread and how disruptive the effects could be. The palpable impact of SARS was translated into action in the form of pandemic influenza planning and surveillance efforts, a greater focus on global health security, improved laboratory and surveillance networks, and most important, the revision of the International Health Regulations (IHR). These regulations had last been updated in 1969, and the experiences with SARS contributed to the urgency to finish the revision. The updates were completed in 2005, when 194 WHO member states approved the international treaty; IHR 2005 went into effect in 2007 (*23*).

The legacy of SARS is evident in many other efforts as well. New national public health agencies have been created in Canada (Public Health Agency of Canada) and the United Kingdom (Health Protection Agency). The WHO Global Outbreak Alert and Response Network has been strengthened (*24*). The Global Disease Detection Program was established at CDC, with the aim of strengthening countries’ efforts in training, surveillance, and outbreak response and establishing 10 Regional Centers by 2012 (www.cdc.gov/globalhealth/gdder) in alignment with the directive for bilateral collaboration and assistance under article 44 of the IHR. With the support of the Bill and Melinda Gates Foundation, the International Association of National Public Health Institutes has been created and now has >75 members around the world (*25*). The Training Programs in Epidemiology and Public Health Interventions Network has expanded, and its regional partners (e.g., African Field Epidemiology Network, Eastern Mediterranean Public Health Network) have been strengthened (*26*).

Perhaps the most important legacy of SARS is the recognition of the critical need for a multilateral response, led by WHO, in the event of a rapidly moving but ultimately containable global epidemic. The central role of WHO in coordinating the laboratory network that identified the etiologic agent and shared reagents, the epidemiology network that characterized the spread and identified the most effective control measures, and the policy and communications network that incorporated rapidly changing knowledge into measured travel advisories was critical for the control of the epidemic and a credit to WHO.

As the importance of cross-species transmission in disease emergence has been increasingly recognized (*27*,*28*), the One Health movement, which emphasizes the importance of interdisciplinary collaboration to address issues at the interface of human health, animal (both domestic and wildlife) health, and environmental/ecosystem health, has gained momentum (*29*,*30*). The US Agency for International Development has supported the Emerging Pandemic Threats Program in an effort to strengthen prediction, detection, response, and amelioration programs in parts of the world shown to be at particular risk (e.g., areas of rainforest intrusion, environmental degradation, ecosystem disruption) for emergence of new diseases (*31*,*32*). The White House recently released the first National Strategy for Biosurveillance, which calls for an all-hazards approach, focusing on threats affecting humans, animals, or plants, to achieve early detection and situational awareness to enable better decision making (*33*).

## Looking Forward

Although many disease detection and control improvements have been implemented in the past 10 years, important gaps in global capacity and coordination remain. One example is the need to greatly strengthen and monitor the national capacity required for full compliance with IHR 2005, including ensuring that adequate numbers of trained personnel are available to support the response to a public health emergency, that surveillance systems are capable of detecting public health emergencies, that access is adequate to laboratory diagnostic capabilities that can identify a range of emerging epidemic pathogens, and that countries have adequate rapid response capacity for public health emergencies (*34*). In addition, for state of the art, affordable countermeasures are needed (especially point-of-care diagnostics, the reinvigoration of the development pipeline for new antimicrobial drugs, and new and improved vaccines), and workable approaches must be determined for equitable distribution of countermeasures when emergencies arise. Finally, systems are necessary to facilitate the conduct of research to evaluate treatment options during public health emergencies, as are tools to assess the utility of social media in strengthening capacity for disease surveillance, event detection, and situational awareness.

Of 194 WHO member states that signed on to the IHR 2005, <20% had achieved compliance with the core capacities required by the deadline of June 2012 (*35*). Assessment of the 13 factors contributing to disease emergence and reemergence identified by IOM expert committees in consensus studies of emerging infections and microbial threats in 1992 (*36*) and 2003 (*2*) suggests that several of these factors contributed to the SARS pandemic (Table). Recent trends for most of these factors continue to operate in favor of the microbes, a finding that indicates a need to identify and respond to other microbial threats (e.g., avian influenza strains, novel CoVs, multidrug-resistant organisms) and emphasizes the necessity for all countries to continue to work on strengthening core capacities for surveillance and response and those for minimizing the risk of cross-border spread (*23*). As we reflect on the lessons learned from the global SARS outbreak, we need to avoid complacency; strengthen efforts to improve global capacity to address the next pandemic using all available 21st century tools; and support research to develop new options, countermeasures, and insights (*37*). At the same time, we must strive to address the global inequities that are the root cause of many of these challenges. 

**Table:** Factors contributing to the emergence of infectious diseases according to IOM reports, 1992 and 2003

1992 IOM Report	2003 IOM Report
**Human demographics and behavior**	Human susceptibility to infection
**Technology and industry**	Climate and weather
**Economic development and land use**	**Changing ecosystems**
**International travel and commerce**	Poverty and social inequality
**Microbial adaptation and change**	War and famine
**Breakdown of public health measures**	**Lack of political will**
	Intent to harm

*Boldface indicates factors that contributed to the emergence and spread of severe acute respiratory syndrome. IOM, Institute of Medicine.
